# A synergistic blocking effect of Mg^2+^ and spermine on the inward rectifier K^+^ (Kir2.1) channel pore

**DOI:** 10.1038/srep21493

**Published:** 2016-02-12

**Authors:** Chiung-Wei Huang, Chung-Chin Kuo

**Affiliations:** 1 Department of Physiology, National Taiwan University College of Medicine, Taipei, Taiwan; 2 Department of Neurology, National Taiwan University Hospital, Taipei, Taiwan

## Abstract

Inward rectifier K^+^ channels (Kir2.1) exhibit an extraordinary rectifying feature in the current–voltage relationship. We have previously showed that the bundle–crossing region of the transmembrane domain constitutes the crucial segment responsible for the polyamine block. In this study, we demonstrated that the major blocking effect of intracellular Mg^2+^ on Kir2.1 channels is also closely correlated with K^+^ current flow, and the coupled movements of Mg^2+^ and K^+^ seem to happen in the same flux–coupling segment of the pore as polyamines. With a preponderant outward K^+^ flow, intracellular Mg^2+^ would also be pushed to and thus stay at the outermost site of a flux–coupling segment in the bundle–crossing region of Kir2.1 channels to block the pore, although with a much lower apparent affinity than spermine (SPM). However, in contrast to the evident possibilities of outward exit of SPM through the channel pore especially during strong membrane depolarization, intracellular Mg^2+^ does not seem to traverse the Kir2.1 channel pore in any case. Intracellular Mg^2+^ and SPM therefore may have a synergistic action on the pore–blocking effect, presumably via prohibition of the outward exit of the higher–affinity blocking SPM by the lower–affinity Mg^2+^.

Inward rectifier K^+^ channels (e.g., Kir2.1 channel) conduct K^+^ ions inwardly through the cell membrane much more efficiently than outwardly^1^,^2^,^3^,^4^,^5^,^6^. Intracellular Mg^2+^ and spermine (SPM) are the key blockers directly modulating ion permeating through the WT Kir2.1 channel in physiological conditions^7^,^8^,^9^,^10^,^11^. We have characterized the biophysical attributes as well as molecular substrates underlying the flow–dependent block of outward K^+^ currents by intracellular SPM, showing that the bundle–crossing region is responsible for the flux–coupling blocking phenomena of SPM and thus constituting the major mechanism underlying inward rectification^12^,^13^. Moreover, SPM may induce gating–like conformation changes in the bundle–crossing region of the pore^12^,^13^. However, relevant attributes of Mg^2+^, the other major physiological pore–blocker of the channel, have remained largely uncharacterized. It has been reported that 0.5–1 mM Mg^2+ ^8,14, and 1–20 μM SPM^8^,^14^,^15^,^16^,^17^ at physiological concentrations both have qualitatively similar flow– and voltage–dependent blocks of the Kir2.1 channel^4^,^8^,^9^,^10^,^11^,^15^,^18^,^19^,^20^,^21^,^22^,^23^. It has also been shown that I176 to A184 residues constituting the bundle–crossing region might also be involved in the blocking effect of SPM on the Kir2.1 channel^24^,^25^. On the other hand, S165, a residue in the transmembrane domain marking the external end of the central cavity in the Kir2.1 channel, is reportedly crucial to intracellular Mg^2+^ but not SPM block^26^. Moreover, it is always an intriguing, but unanswered question what is the actual role of Mg^2+^ in the molecular physiology of Kir2.1 channels, given the fact that the coexisting polyamines (e.g., SPM) are already very potent voltage– and flux–dependent pore blockers effectively making the inward rectification phenomenon. It is therefore desirable to study the biophysical as well as molecular attributes of Mg^2+^ block Kir2.1 channel pore in more detail, and to make a comparison with SPM to decipher the differential and combinational roles of these two physiological blockers of Kir2.1 channel pore.

In this study, we demonstrate that the blocking effect of intracellular Mg^2+^ on the Kir2.1 channel is correlated with K^+^ currents flow, albeit quantitatively much less marked than SPM. The coupled movement of Mg^2+^ and K^+^ ions also happens in the same flux–coupling pore segment at the bundle–crossing region as SPM. With preponderant outward K^+^ flow, Mg^2+^ is pushed to the outermost site of the flux–coupling segment in the bundle–crossing region of the Kir2.1 channel pore. Because Mg^2+^ is much less likely to exit to the outside upon very strong depolarization or preponderant outward flow than SPM, Mg^2+^ may effectively enhance the blocking effect of SPM, a blocker with an apparently much higher affinity than Mg^2+^ upon moderate depolarization, but an evident tendency of outward exit of the pore with large driving forces.

## Results

### Inhibition of WT Kir2.1 currents by intracellular Mg^2+^ in symmetrical 100 mM K^+^ solution

**Figure 1:**
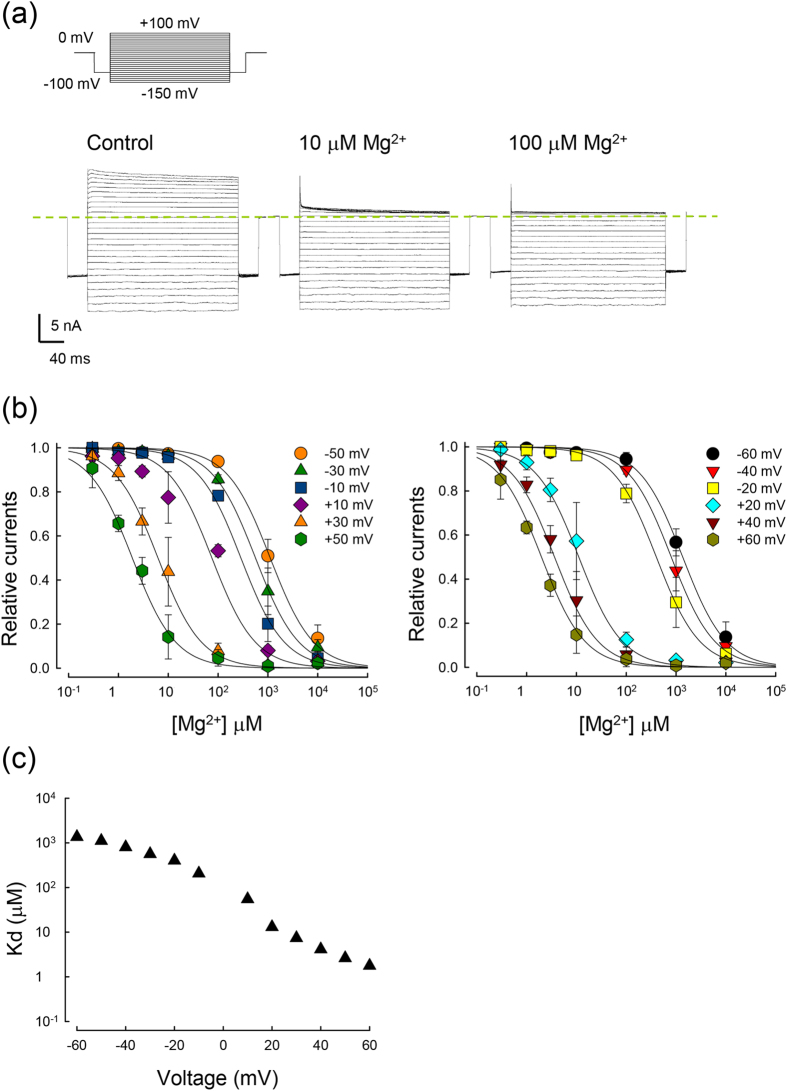
The affinity of intracellular Mg^2+^ for WT Kir2.1 channel in symmetrical 100 mM K^+^. (**a**)Sample sweeps demonstrating the inhibition of WT Kir2.1 currents in the same patch by intracellular Mg^2+^ in symmetrical 100 mM K^+^. The membrane voltage was repolarized from a holding potential of 0 mV to −100 mV for 20 ms, and then stepped to test pulses between −150 and +100 mV in 10 mV increment for 150 ms. The dashed lines indicate the zero current level. (**b**) The relative current is defined by the ratio between the steady–state currents in Mg^2+^ and in control at each designated membrane potential, and are plotted against Mg^2+^ concentration (n = 5–8). The data obtained at different membrane voltages (from −60 to +60 mV) are fitted by Eq. 1 (see Methods). (**c**) The Kd from part **b** is plotted against membrane voltage on a semi–logarithmic scale. The Kd–voltage relationship is clearly nonlinear, with the most evident change around 0 mV (the reversal potential of K^+^ ion; E_K_^+^). The IR index (defined as the ratio between the Kd values at −30 and +30 mV^13^) is ~95 in this case.

We first examined the flow–dependence of the intracellular Mg^2+^ block of the WT Kir2.1 channel. Figure 1a,b, shows the inhibition of macroscopic WT Kir2.1 currents in symmetrical 100 mM K^+^ solution by 10 and 100 μM intracellular Mg^2+^. The outward currents are inhibited by intracellular Mg^2+^ with the decay phase accelerated in a dose–dependent manner. The inward currents, on the other hand, are not evidently affected by intracellular Mg^2+^. Figure 1c shows that the apparent Kd of Mg^2+^ has relatively prominent changes in the vicinity of 0 mV (the reversal potential of K^+^ ions; E_K_^+^). The change, however, is far less abrupt if compared to the case of spermine (SPM) examined in a similar way^12^. One may take the ratio between Kd values of −30 to +30 mV as a quantitative measurement of the inward rectification feature of the block (i.e., “IR index”) which is ~95 here for Mg^2+^ but is ~102,000 in the case of SPM^12^. We also examined the effect of intracellular Mg^2+^ and SPM on non–Kir2.1 channel expressing membranes in symmetrical 100 mM K^+^ as a control. There is no discernible effect on the non–Kir2.1 channel expressing membranes whether Mg^2+^ and SPM are present alone or concomitantly (supplementary Figure S1).

### Competition between permeating K^+^ and blocking Mg^2+^ in the WT Kir2.1 channel pore

**Figure 2:**
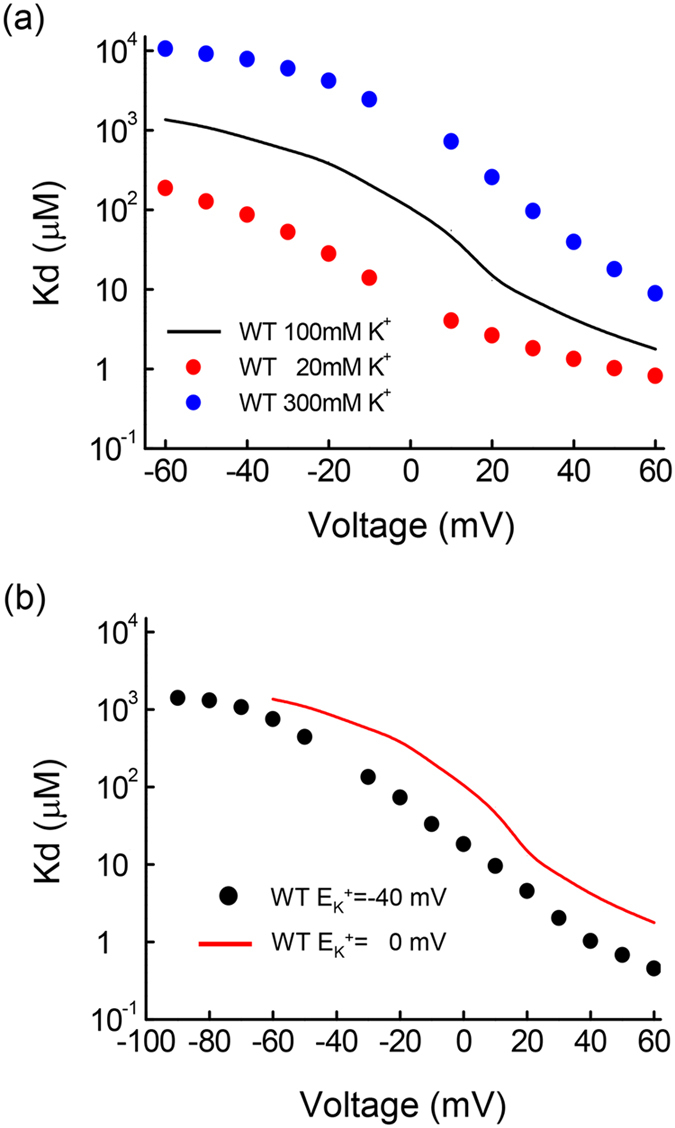
Inhibition of WT Kir2.1 currents by intracellular Mg^2+^ in symmetrical 20 mM K^+^, 300 mM K^+^, and external 20/ internal 100 mM K^+^. (**a**)The Kd of the intracellular Mg^2+^ block in symmetrical 20 and 300 mM K^+^ is plotted against voltage on a semi–logarithmic scale (data were obtained with the same approach as that in Fig. 1b). The Kd–voltage relationship of Mg^2+^ block in symmetrical 100 mM K^+^ (solid line) is taken from Fig. 1c for comparison. (**b**) The Kd–voltage relationship of Mg^2+^ block in external 20/internal 100 mM K^+^ is apparently similar to that in symmetrical 100 mM K^+^, but is shifted only by ~−20 mV in the voltage axis (data were obtained with the same approach as that shown in Fig. 1c).

We subsequently changed the ambient K^+^ and repeated the experiments symmetrical 20 or 300 mM K^+^ solution. The same as the case in symmetrical 100 mM K^+^ solution, the apparent Kd of Mg^2+^ in symmetrical 20 and 300 mM K^+^ solutions shows a relatively abrupt change around 0 mV (E_K_^+^) (Fig. 2a). Interestingly, the Kd–voltage relationship is roughly of the same shape in symmetrical 100 and 300 mM, but is apparently shallower in 20 mM symmetrical K^+^. Moreover, it is evident that the apparent Kd of Mg^2+^ is higher with higher ambient K^+^ concentration. These results are consistent with the idea that permeating K^+^ ions could “abduct” the blocking Mg^2+^ to move in the same direction, and that K^+^ and Mg^2+^ may compete for the same site(s) in the flux–coupling region. These flux coupling sites, but not the blocking site itself, thus seem to be saturated by 100–300 but not 20 mM ambient K^+^. We further investigated the effect of the intracellular Mg^2+^ with a E_K_^+^ of −40 mV. The binding constant (Kd) of the Mg^2+^ block in asymmetrical (external 20/internal 100 mM) K^+^ is shifted by only ~−20 mV (Fig. 2b). This is an evidently smaller value than the shift examined in a similar way for SPM^12^. Together with the much smaller IR index for Mg^2+^ than SPM in Fig. 1, these findings indicate that the flux–coupling effect is much stronger for SPM than Mg^2+^ block of the Kir2.1 channel pore.

### A substantial decrease of the rectification feature and emergence of a slow unblocking phase of Mg^2+^ in specific E224 and E299 mutant channels

**Figure 3:**
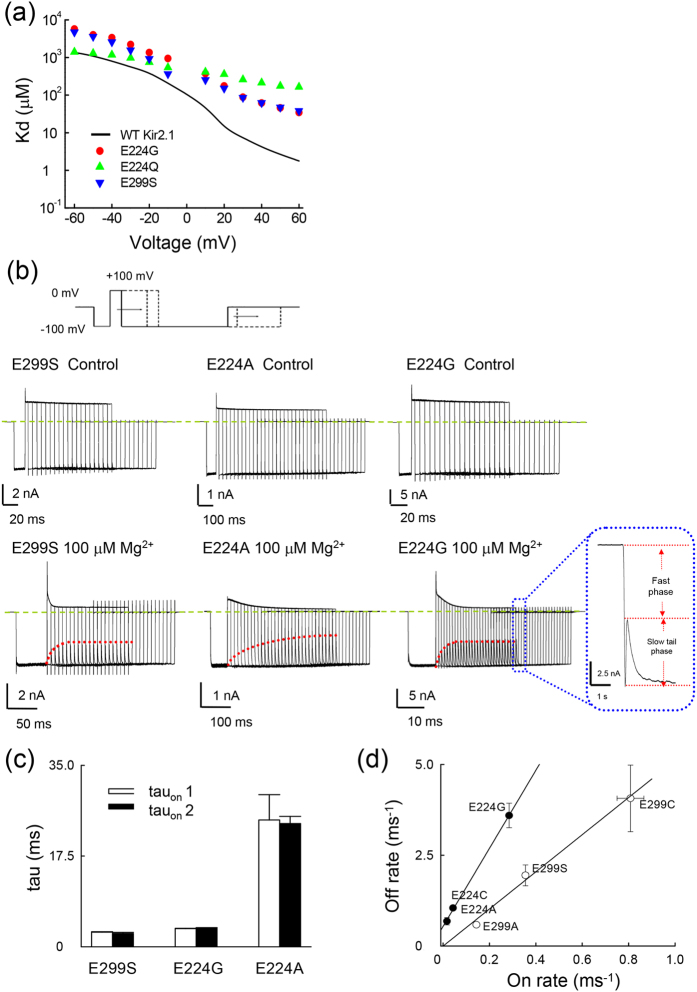
The emergence of a slow component of intracellular Mg^2+^ unblock in mutant E224 and E299 channels. (**a**) The Kd of intracellular Mg^2+^ block in E224G, E224Q, and E299S mutant channels is plotted against voltage on a semi–logarithmic scale (the same approach as that shown in Fig. 1c). The changes in Kd of intracellular Mg^2+^ block around 0 mV are evidently much smaller with specific of E224G, E224Q, and E299S mutations. (**b**) The patches which expressed E299S, E224G, and E224A mutant channels were first held from 0 mV and stepped twice to −100 mV^12^,^13^,^27^. The sample sweeps show a “slow tail” when the patch was repolarized from a positive voltage of +100 mV to a negative voltage of −100 mV in the presence but not in the absence of intracellular Mg^2+^, signaling Mg^2+^ block from the channel. The development of the slow tail in the second −100 mV pulse thus is used as a measure of intracellular Mg^2+^ binding to this slow unblocking site at +100 mV (dashed lines, see also part **c**). The dashed lines indicate the zero current level. Note that when the currents are turning from outward to inward, there is a very fast phase of development of inward current before the slow tail phase (insert figure). (**c**) Cumulative results were obtained with the same protocol described in part **b** for the E299S, E224G, and E224A mutant channels (n = 4–7). tau _on, 1_ and tau _on, 2_ denote the time constants of the growing courses of slow tails and the time constants of the decay of outward currents in the presence of 100 μM intracellular Mg^2+^ in part **b** respectively. (**d**) The reciprocals of the time constants of development of slow tail currents (on rate at +100 mV) are plotted against the decay time constant of the slow tail (off rate at −100 mV) for each of the E224 mutant (E224A, E224C, and E224G) and E299 mutant (E299A, E299S, and E299C) channels. The lines are linear regressions demonstrating the strong linear correlation between the two parameters.

The E224 and E299 residues in the cytoplasmic domain have been reported to bear a close association with the function of the Kir2.1 channel^12^. We found that the apparent Kd of Mg^2+^ at different membrane potentials is also larger in the E224G, E224Q, and E299S mutant channels than that in the WT channel (Fig. 3a). Moreover, the Kd–voltage plot no longer shows a substantial alter around the reversal potential of K^+^ ions in these mutant channels. This finding implicates that E224 and E299 residues in the cytoplasmic domain also play a critical role in the making of the flux–dependent Mg^2+^ block of the Kir2.1 channel. Nevertheless, there is a “slow tail” in the intervening −100 mV pulse in these mutant channels in the presence of intracellular 100 μM Mg^2+^, but not in the control condition (Fig. 3b). The slow tail is apparently ascribable to the slow unblocking of Mg^2+^ from a blocking site^12^,^13^,^27^. Also, the slow tail exhibits the same decay time constant irrespective of length at the +100 mV pulse. The developmental course of the slow tail, therefore, may signal the course of Mg^2+^ blocking to a single slow unblocking site at the preceding pulse at +100 mV, and consequently shares the same time course with the decay of the outward currents at +100 mV (Fig. 3c). These results imply that the intracellular Mg^2+^ might be pushed to the outermost or innermost binding site by outward or inward K^+^ currents, respectively^12^ (see DISCUSSION for more details). We also performed other substitutions (A, C, N, W, S, and R) for E224 and E299 sites, and obtained four additional mutant channels in E224 and E299 sites (E224A, E224C, E299A, and E299C) giving rise to enough currents for analysis. Figure 3d further shows that the kinetics of the development and those of the decay of the slow tail are closely correlated with each other in all of these E224 and E299 mutant channels. These findings indicate that E224 and E299 mutations most likely induce a pore–narrowing change in the flux–coupling segment to correlatively slow down the blocking and unblocking kinetics of (intracellular) Mg^2+^. These features are very similar to SPM^12^,^13^, and thus strongly implicate that Mg^2+^ shares the same flux–coupling segment of the channel pore as SPM.

### Lack of voltage dependence of the blocking kinetics but strong voltage dependence of the unblocking kinetics of intracellular Mg^2+^

**Figure 4:**
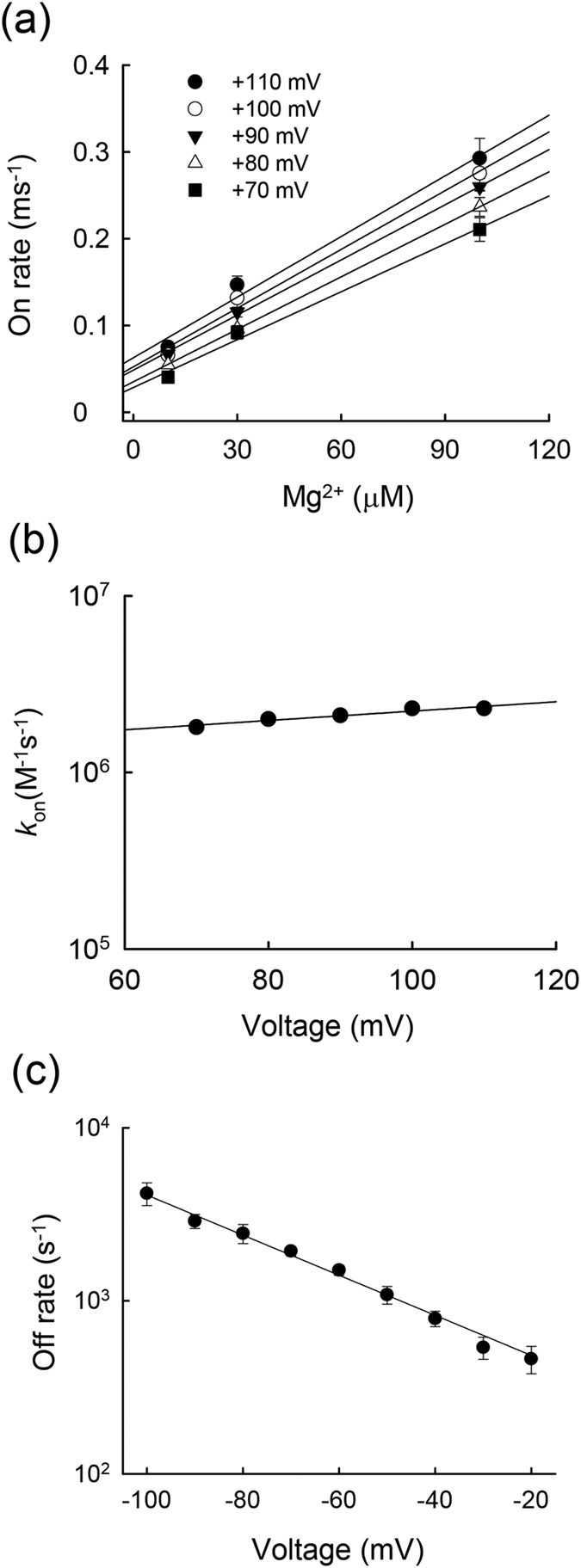
The blocking and unblocking kinetics of internal Mg^2+^ in E224G mutant channels. (**a**) The inverses of the time constants for the development of the slow tail in Fig. 3c are plotted against the Mg^2+^ concentration (the depolarization pulses were set between +70 and +110 mV). The lines are linear fits with slope and Y–intercept of 1.8 × 10^6^ M^−1^s^−1^ and 29 s^−1^ (+70 mV); 2 × 10^6^ M^−1^s^−1^ and 35 s^−1^ (+80 mV); 2.1 × 10^6 ^M^−1^s^−1^ and 49 s^−1^ (+90 mV); 2.3 × 10^6 ^M^−1^s^−1 ^and 52 s^−1^ (+100 mV); and 2.3 × 10^6 ^M^−1^s^−1^ and 63 s^−1^ (+110 mV), respectively. (**b**) The slopes in part **a** are plotted against voltage and fitted with the equation: *k*_on_ = 1.2 × 10^6^ exp (0.15V/25 mV) M^−1^s^−1^, where V is the membrane voltage potential in mV. The regression line indicates an equivalent electrical distance (δ) of ~0.08 for the Mg^2+^ blocking rate (assuming +2 effective charges on Mg^2+^).(**c**) The inverses of the relaxation time constants of the slow tail currents are plotted against membrane potential on a semi–logarithmic scale for the E224G mutant channels (n = 5). The second negative pulse in the same two–pulse protocol was set between −20 and −100 mV to examine the voltage dependence of Mg^2+^ unblock from the blocking site. The data are fitted with the equation: off rate _(V)_ = off rate _(0)_ × exp(–ZδV/25 mV), where V is the membrane potential in mV, and Z and δ are the charges on the blocker and the equivalent electric distance of the blocking site from inside, respectively. The off rate_(0)_ and Zδ are ~280 s^−1^ and ~0.67 for the E224G mutant channels.

In our previous study, we demonstrated that intracellular SPM block of the Kir2.1 channel is composed of a strongly voltage–dependent unblocking (δ = ~0.58) and a voltage–independent (δ = ~0.06) blocking processes^12^. Intriguingly, the blocking rate of Mg^2+^ to this blocking site is also barely voltage–dependent (δ = ~0.08; Fig. 4a,b), whereas the unblocking rate is strongly voltage–dependent (δ = ~0.33, assuming two effective charges on Mg^2+^; Fig. 4c). Because the voltage dependence of on and off rates was obtained with preponderant outward (~+70 to +110 mV) and inward currents (~−20 to −100 mV), respectively, the foregoing voltage dependence should be essentially devoid of the flux–coupling effect, and thus provide a rather accurate measurement of the location of the Mg^2+^ blocking site. The similar patterns of the voltage–dependence of blocking and unblocking rates would strongly suggest that intracellular Mg^2+^ and SPM cross the same barrier to reach the flux–coupling region in the pore. The relative faster blocking and unblocking rates of Mg^2+^ than SPM, on the other hand, may signal that it is more difficult to move a molecule bearing sequential changes across a narrow pore segment without sequentially arranged counterparts of “receptor” sites (e.g., sites of counter charges or opposite polarity).

### Similar voltage dependence of the intracellular Mg^2+^ unblocking kinetics in the WT and different mutant channels

**Figure 5:**
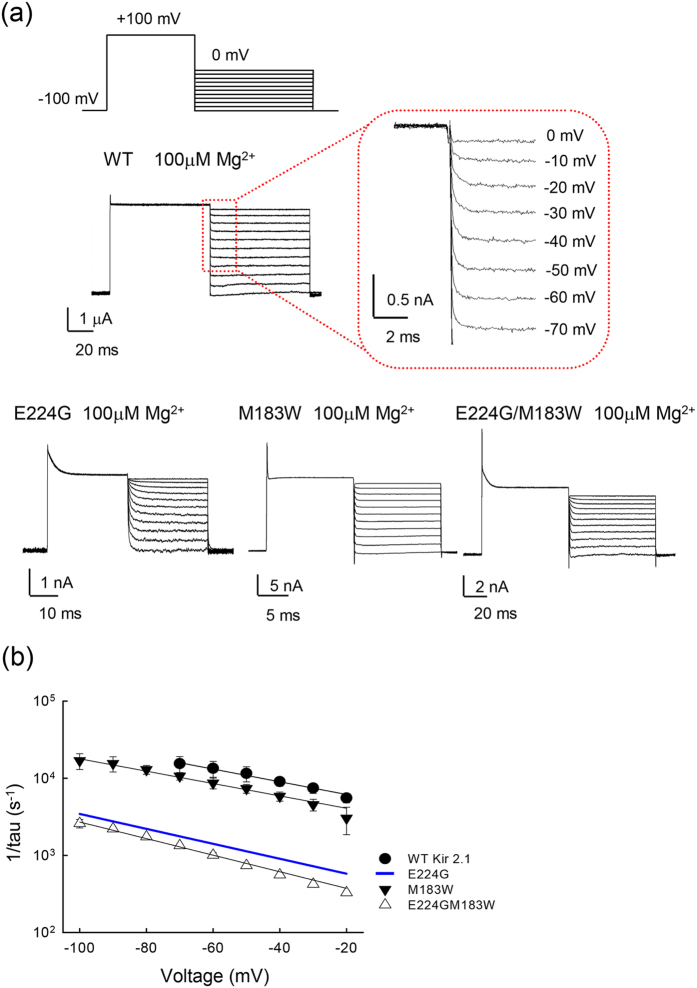
Similar voltage dependence of the intracellular Mg^2+^ unblocking kinetics in the WT, E224G, and M183W mutant channels. (**a**) The two–pulse protocol is basically similar to that in Fig. 3b, except that the voltage across the patch membrane was first depolarized from the holding potential −100 mV to +100mV for ~50 ms, and then stepped to different test voltages between −100 and 0 mV for ~300 ms in 10 mV increment for M183W single, and E224G/M183W double mutant channels. Representative current traces were recorded in control or in internal 100 μM Mg^2+^. The slow tail is present only in the presence of intracellular Mg^2+^ at appropriate membrane potentials. (**b**) The reverses of the relaxation time constants of the slow tail currents are plotted against voltage in semi–logarithmic scale for the WT, and single as well as corresponding double mutant channels. The negative pulse in the same protocol was set to −20 ~ −100 mV to examine the voltage dependence of Mg^2+^ unblock from the binding site. The E224G mutant channel data are taken from Fig. 4c for comparison (the blue line). The off rate_(0)_ and Zδ are ~4300 s^−1 ^and 0.48 for the WT channel, ~370 s^−1^ and 0.56 for the E224G, ~2800 s^−1^ and 0.46 for the M183W, ~230 s^−1^ and 0.62 for the E224G/M183W mutant channels, respectively.

Figure 5a shows that the slow tail due to Mg^2+^ unblocking is discernible not only in mutant, but also in the WT Kir2.1 channels at “appropriate” voltage ranges. Figure 5b summarizes the unblocking rates of intracellular Mg^2+^ in the WT and different mutant channels (single and double mutations involving E224 and M183). The monotonous deceleration at more positive membrane voltages indicates slower unblocking rates of the blocking Mg^2+^ with membrane depolarization. Despite quite evident variations in the absolute values of the off rates, the voltage dependence remained similar among the WT and different mutant channels, suggesting a significantly altered height but not location and shape of the major barrier to the blocking Mg^2+^ exiting the pore. These data are very similar to SPM^12^, and are consistent with the view that mutation of E224 and at least residues M183 change the height of the same barrier for Mg^2+^ permeation between the blocking site and intracellular melieu.

### Pore–narrowing effects at the bundle–crossing region of the transmembrane domain in the Kir2.1 channel by E224 mutations

**Figure 6:**
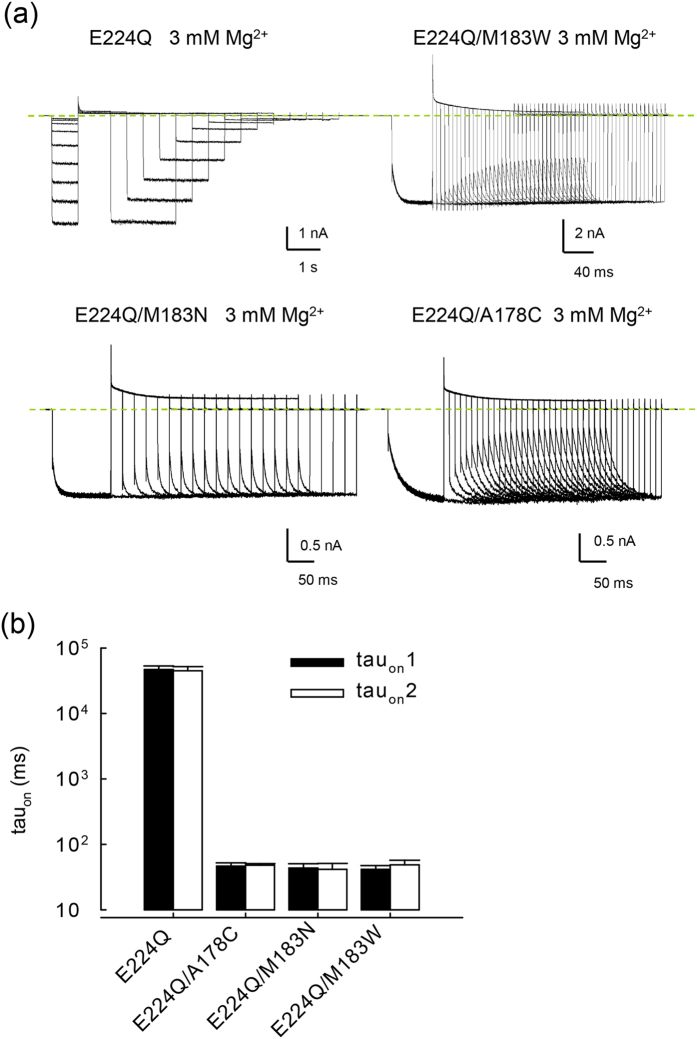
Elimination of the extremely slow component of Mg^2+^ unblock in E224Q mutant channel by concomitant specific mutations at M183 and A178. (**a**) Sample sweeps of the extremely slow tail currents in the E224Q mutant channel in symmetrical 100 mM K^+^ (see Fig. 3c for the pulse protocol). The slow tails are considerably slower than that in the E224G mutant channel. Also, much higher concentrations of Mg^2+^ are required for the emergence of slow tails. In addition, the extremely slow tails signaling Mg^2+^ unblocking in the E224Q single mutant channel are evidently accelerated in specific double–mutant (e.g., E224Q/M183W, E224Q/M183N, and E224Q/A178C) channels. The dashed lines indicate the zero current level. (**b**) Cumulative results were obtained for the E224Q, E224Q/M183W, E224Q/M183N, and E224Q/A178C double mutant channels (each n = 5). tau _on, 1_ and tau _on, 2_ denote the time constant of the growing course of the slow tails (at −100 mV) and the time constant of the decay of the macroscopic outward currents (at +100 mV) in 3 mM Mg^2+^ from the experiments in part **a** respectively (the same approach as that shown in Fig. 3c).

Because E224 is located in the cytoplasmic domain, a widened part of the Kir2.1 channel pore, we tried to define the actual location of the pore–narrowing region in E224 mutant channels with concomitant mutations. We found that the extremely slow blocking and unblocking processes of intracellular Mg^2+^ in the E224Q mutant channel are substantially accelerated by concomitant mutations at the bundle–crossing regions of M183W, M183N, and A178C (Fig. 6a). Also, we examined the decay of macroscopic outward currents at +100 mV in the E224Q/M183W, E224Q/M183N, and E224Q/A178C double–mutant channels. The decay of outward currents at +100 mV again is very similar to the development of slow tails in these double–mutant channels (Fig. 6b). These findings collectively indicate that the E224Q mutation changes the pore size at the bundle–crossing region of transmembrane domain (TM2) (e.g., M183 and/or A178), and thus the blocking/unblocking kinetics of intracellular Mg^2+^. These data are once more similar to the case of intracellular SPM block of the E224G mutant channel^12^.

### Flow–dependence and inward rectification of intracellular Mg^2+^ block at the bundle–crossing region in the Kir2.1 channel pore

**Figure 7:**
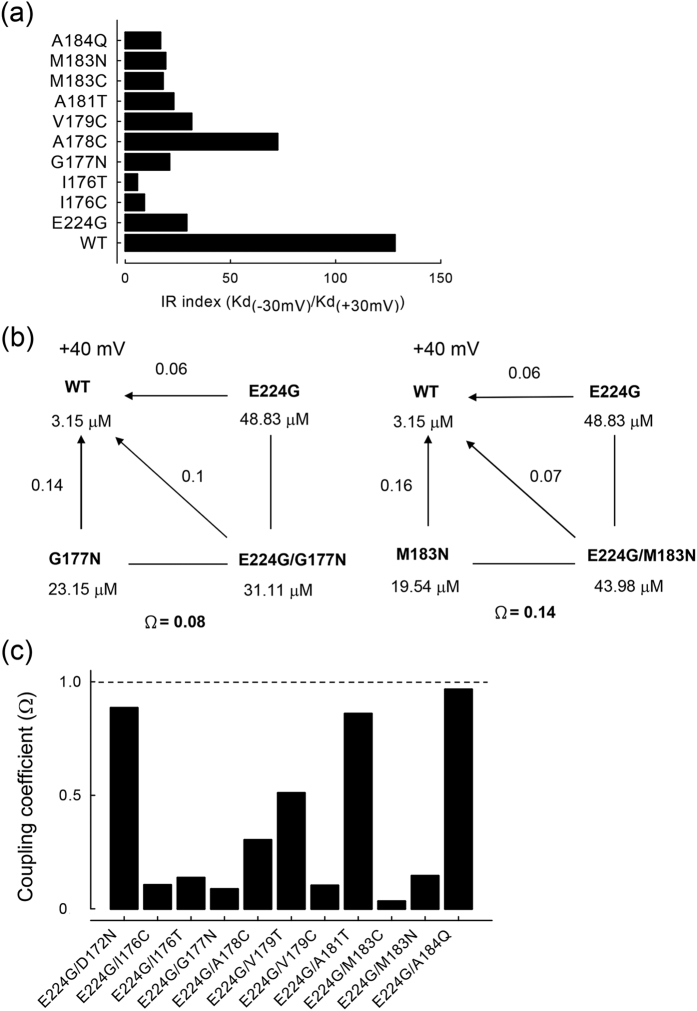
Negative cooperative effects of E224G and the TM2 bundle–crossing region mutations on the apparent affinity of Mg^2+^. (**a**) Comparison of the ratio between the apparent Kd of intracellular Mg^2+^ at −30 and +30 mV among the WT and mutant channels. (**b**) Double–mutant cycle analysis of the E224G+G177N and E224G+M183N single– and double–mutant channels at +40 mV. The Kd of Mg^2+^ block is derived from the same approaches as that described in Fig. 1c. The coupling coefficients (Ω) are defined as the ratio between the product of the Kd changes in each single mutant channel (versus the WT channel) and the Kd change in the double–mutant channel, and are ~0.08 and ~0.14 for the E224G/G177N and the E224G/M183N double mutations, respectively. (**c**) The coupling coefficient is plotted for different pairs of double mutations. The dashed line denotes a coupling coefficient of 1.0, which signals a purely additive effect of the paired mutations. Double mutations involving E224 (E224G) and most of the residues between I176 and M183 in the bundle–crossing region of TM2 show a prominent negative cooperative effect, except for the E224G/A181T double mutant channels. E224G/D172N and E224G/A184Q double mutations involving E224 and presumably either the outer or the inner boundaries of the central cavity, also have coupling coefficients close to ~1.0.

To further examine the role of the residues located immediately between I176 and M183, we constructed a series of single mutations from I176 to M183, which presumably constitute the bundle–crossing region of TM2. Figure 7a shows a marked decrease in the inward rectification features of internal Mg^2+^ block in the I176C, I176T, G177N, A178C, V179C, A181T, M183C, and M183N mutant channels, quite similar to the case of the E224G mutant channel. These results strongly substantiate that the bundle–crossing region I176–M183 is involved in the mechanism underlying the inward rectification of the intracellular Mg^2+^ block in the Kir2.1 channel. Figure 7b shows that both E224G/G177N and E224G/M183N double mutations display strong negative cooperative effects on the apparent Kd of Mg^2+^. A similar effect could be observed for the double mutations involving E224G and most of the residues in the bundle–crossing region of TM2 (Fig. 7c). E224G and the bundle–crossing region of TM2 (I176–M183) therefore act not only at the same major energy barrier for Mg^2+^ permeation (from the intracellular melieu to the blocking site), but also at the same blocking site itself. In other words, although mutations involving E224 and the bundle–crossing region of TM2 are separated by >40 residues in the amino acid sequence, they seem to share the same loci of action for intracellular Mg^2+^ block. These features are once more very much reminiscent of that of internal SPM block of the Kir2.1 channel.

### Markedly different actions of Mg^2+^ and SPM in terms of traversing the outmost part of the Kir2.1 channel pore

**Figure 8:**
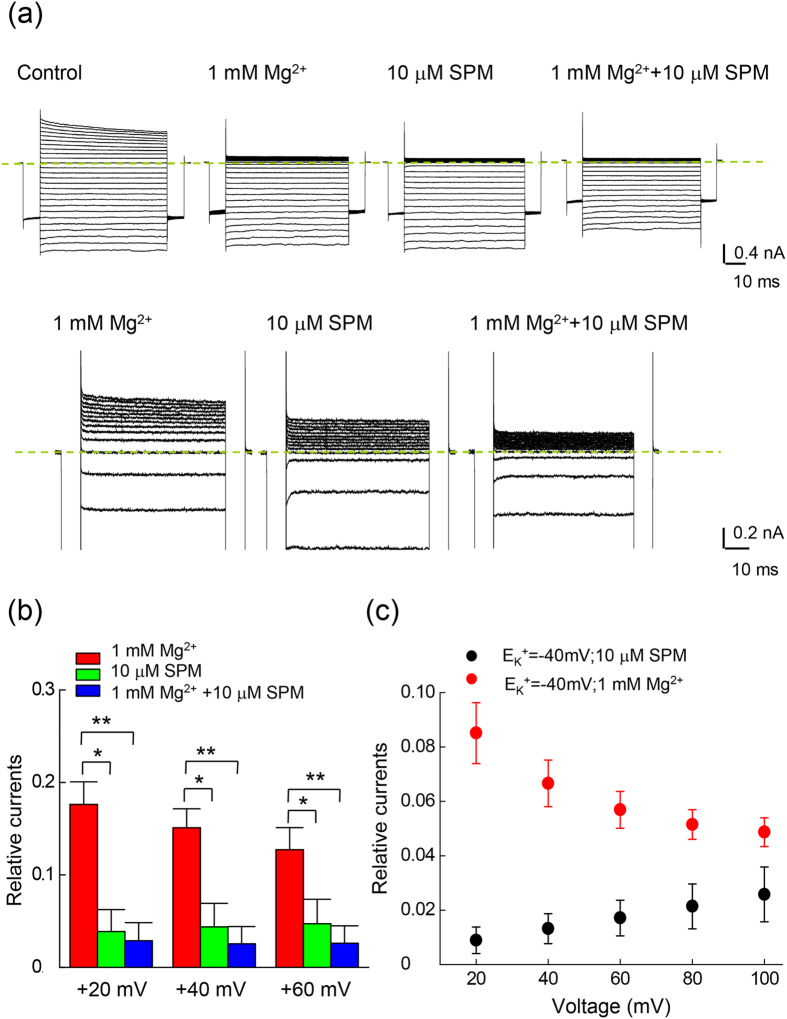
Inhibition of outward currents in the WT Kir2.1 channel by internal 1 mM Mg^2+^ and 10 μM SPM. (**a**) Sample sweeps demonstrating inhibition of WT Kir2.1 currents in the same patch by internal 1 mM Mg^2+^, 10 μM SPM, and concomitant 1 mM Mg^2+^ and 10 μM SPM in symmetrical 100 mM K^+^ (see Fig. 1a for the pulse protocol). The dotted lines indicate the zero current level. (**b**) The experiments were performed in symmetrical 100 mM extracellular K^+^ for the WT Kir2.1 channel. The relative currents are defined in the same way as that in Fig. 1. Note the stronger inhibitory effect (smaller residual currents) with 10 μM SPM than 1 mM Mg^2+^, and the even stronger effect (even smaller residual currents) with the concomitant presence of both 1 mM Mg^2+^ and 10 μM SPM. * and **p < 0.05 and <0.005, respectively, by student’s independent *t*–test. (**c**) The experiments were performed in external 20/ internal 100 mM K^+^ conditions for the WT Kir2.1 channel. The relative currents are defined in the same way as that in Fig. 1. The protocols were the same as that in Fig. 1a. The blocking effect of 1 mM Mg^2+^ is gradually increased, whereas the blocking effect of 10 μM SPM is gradually decreased, from +20 to +100 mV.

In view of the marked qualitative similarity between the molecular actions of Mg^2+^ and SPM on the Kir2.1 channel, it is intriguing why there are the two blockers in the cell. In other word, what would be the role of Mg^2+^ given the already very strong blocking effect of SPM? We examined the blocking effect of coexisting internal Mg^2+^ and SPM. Figure 8 shows that the blocking effect on the outward Kir2.1 currents is much more pronounced with 10 μM SPM than 1.0 mM Mg^2+^, the presumable “physiological concentrations” of the blockers in certain cells^8^,^14^,^15^,^16^,^28^. There are still evident residual outward currents with either 1 mM Mg^2+^ or 10 μM SPM. Concomitant intracellular 1 mM Mg^2+^ and 10 μM SPM apparently could further reduce the residual currents in the presence of either blocker alone. It is interesting that the much weaker blocker (1 mM) Mg^2+^ could be so prominently increase the blocking effect of the much stronger blocker (10 μM) SPM. A closer look at Fig. 8b reveals that the residual currents decrease with increasing depolarization in the case of Mg^2+^ block, but not in the case of SPM block (where there is even a tendency of increasing residual currents) with stronger depolarization. This could be ascribed to the increasing tendency of outward exit (and thus decreased blocking potency) of the blocking SPM, but not Mg^2+^, when there is more and more preponderant outward flux of the permeating K^+^ ion. We therefore did the same experiments in a more physiological condition (external 20 mM K^+^/ internal 100 mM K^+^ condition, E_K_^+^ = −40 mV), which would result in even more preponderant outward K^+^ flux at the same membrane voltage (Fig. 8c). It is evident that the relative currents in the presence of intracellular 1.0 mM Mg^2+^ decrease with increasing membrane depolarization from +20 mV to +100 mV, whereas the relative currents with intracellular 10 μM SPM increase from +20 mV to +100 mV. This would strongly support the view that more of the blocking SPM may exit to the outside at more positive voltages. In contrast, the blocking Mg^2+^ does not have a discernible chance of outward exit to “permeate” through the Kir2.1 channel pore even at extremely depolarized membrane potentials. Intracellular Mg^2+^ and SPM thus no longer exhibit similar but distinct characteristics in terms of traversing the outmost part of the Kir2.1 channel pore.

### There is also an additive kinetic effect between Mg^2+^ and SPM

**Figure 9:**
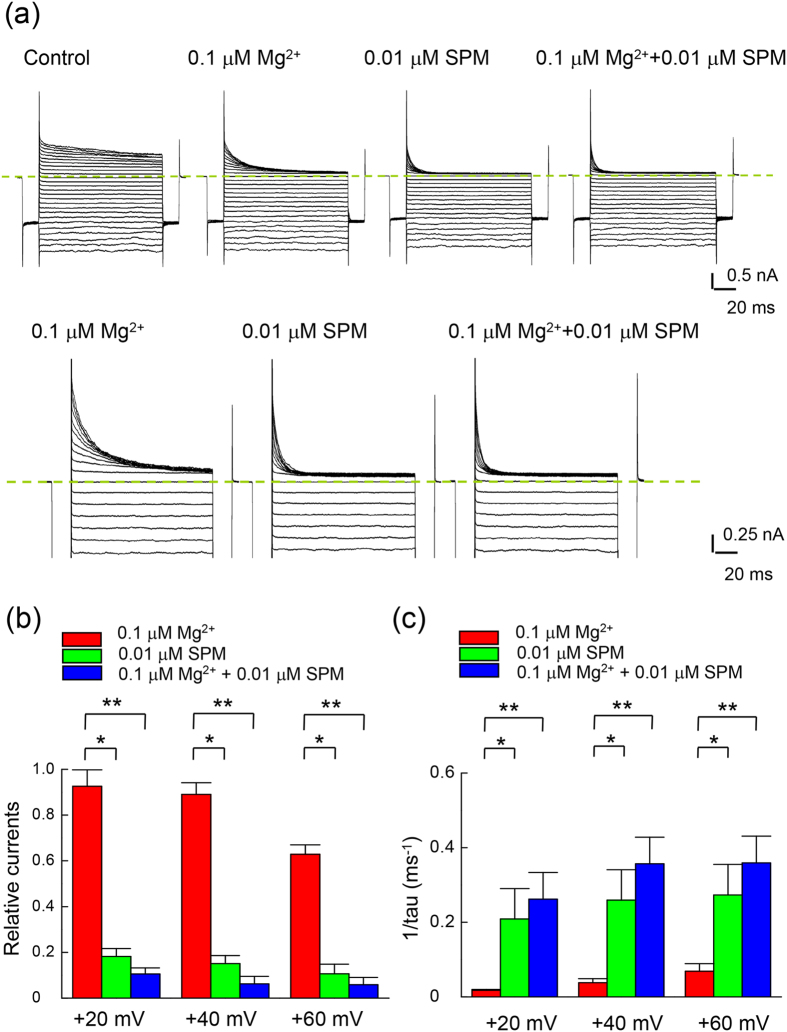
Inhibition of outward currents in the WT Kir2.1 channel by internal 0.1 μM Mg^2+^ and 0.01 μM SPM. (**a**) Sample sweeps demonstrating the inhibition of WT Kir2.1 currents in the same patch by intracellular 0.1 μM Mg^2+^, 0.01 μM SPM, and concomitant 0.1 μM Mg^2+^ and 0.01 μM SPM in symmetrical 100 mM K^+^ (the same pulse protocol as that in Fig. 1a). The dashed lines indicate the zero current level. (**b**) The experiment was conducted in symmetrical 100 mM extracellular K^+^ for the WT Kir2.1 channel. Note the stronger blocking effect in the concomitant presence of both 0.1 μM Mg^2+^ and 0.01 μM SPM (e.g., +20, +40, and +60 mV) than either blocker. * and **p < 0.05 and <0.005, respectively, by student’s independent *t*–test. (**c**) Cumulative results were obtained from experiments with the same protocols described in part **a** (n = 7). Note that the macroscopic binding rates of concomitant intracellular 0.1 μM Mg^2+^ and 0.01 μM SPM are roughly the sum of the binding rates of each blocker alone. * and **p < 0.05 and <0.005, respectively, by student’s independent *t*–test.

**Figure 10:**
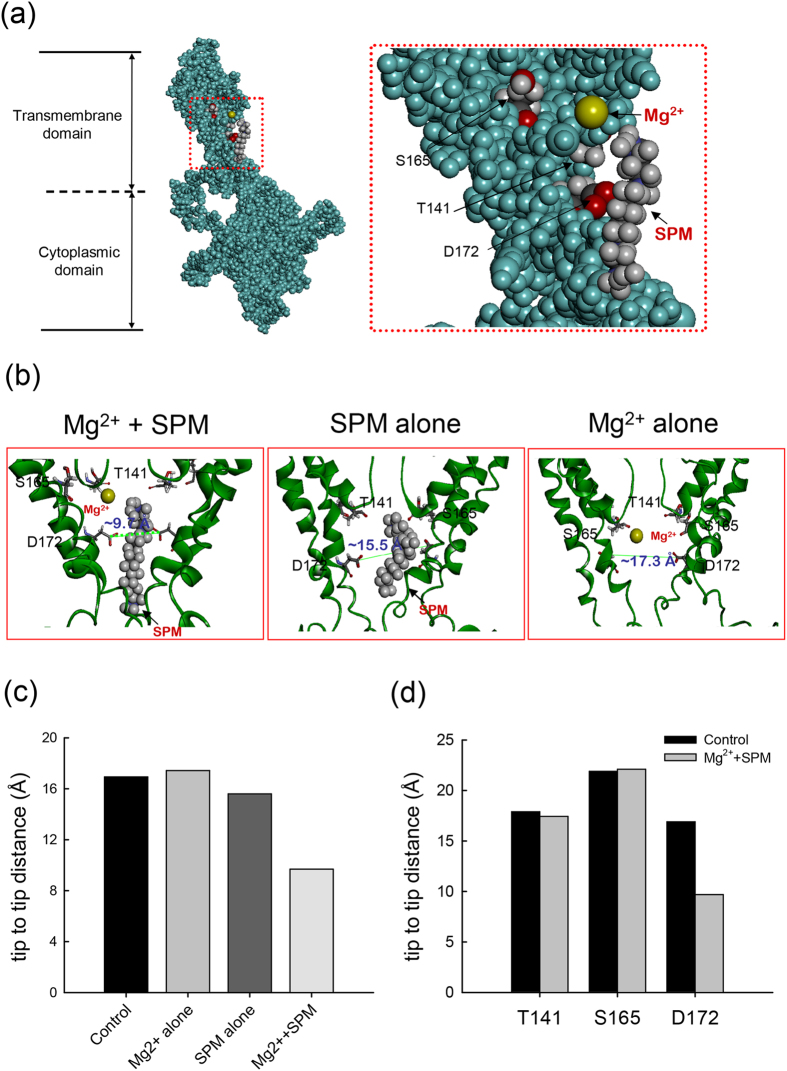
Molecular dynamics simulation of coexistence Mg^2+^ and SPM action in the WT Kir2.1 channel pore. (**a**) In the presence of approximately forty water molecules, one Mg^2+^, one SPM molecules, and seven K^+^ ions are applied to the central cavity region of the WT Kir2.1 channel. The Mg^2+^ (colored are yellow) and SPM molecules (hydrogen atoms are colored grayish white; carbon, black; and nitrogen, blue) of the blocking sites are presented in a CPK model. The representative structure of the cytoplasmic and transmembrane domains of one subunit of the WT Kir2.1 channel is shown. A regional view of the residues T141, S165, and D172 is shown in the boxed panel. The side chains of T141, S165, and D172 are shown in CPK model, and the hydrogen atoms are colored grayish white; nitrogen, blue; oxygen, red; and carbon, black. All of the other atoms in the channel protein are colored green. (**b**) With the same approaches as that in part **a** two subunits of the channel are presented in a solid–ribbon model. The distance between diagonal residues D172 from tip to tip is profoundly decreased (~9.7 Å) in the coexistence presence of Mg^2+^ and SPM, but not in presence of Mg^2+^ (~17.3 Å) or SPM alone (~15.5 Å). Note that Mg^2+^ is located close to S165 whereas SPM is located close to D172. (**c**) A summary plot of the tip–to–tip diagonal distance between sites D172 in control, and in the presence of SPM, or Mg^2+^, or both, in the WT Kir2.1 channel pore. Note that an evident narrowing occurs only in the concomitant presence of Mg^2+^ and SPM. (**d**) A summary plot of the diagonal (tip–to–tip) distances between residues T141, S165, and D172 in control (no blockers) and in the concomitant presence of Mg^2+^ and SPM in the Kir2.1 channel pore. Note that an evident narrowing occurs only at the level of D172, but not at T141 and S165.

We have seen a synergistic action between intracellular Mg^2+^ and SPM in blocking the outward currents through Kir2.1 channels. Despite of its much lower apparent affinity, Mg^2+^ could evidently enhance the blocking effect of SPM by prohibition of the outward exit of the blocking ion. Because of the common nature of the one–to–one binding reaction and the overlapping sites of action between the two ions, the onset of block (i.e. blocking rates) could also be kinetically additive in addition to the foregoing synergism based on a more steady–state consideration. To investigate the interactions during the onset of block, we reduced the concentrations of both blockers to submicromolar range for better resolution of the kinetics (Fig. 9). It is readily discernible that the decay of outward currents gets more rapid in the presence of both than each single blocker (Fig. 9a). The synergistic effect on the steady–state currents remains manifest with such low concentrations of both blockers (Fig. 9b). Most interestingly, the blocking rates are roughly additive with the coexistence of both blockers (Fig. 9c). The physiological role of intracellular Mg^2+^, which is usually present in millimolar concentrations, in the modulation of Kir2.1 channel with concomitant micromolar SPM is thus twofold. At the beginning of the depolarization phase, Mg^2+^ would contribute to an extremely fast decrease of K^+^ effluxes through the Kir2.1 channel. In the later phase of depolarization, on the other hand, Mg^2+^ could further decrease the steady–state K^+^ effluxes chiefly by prohibition of the outward exit of SPM (also see DISCUSSION and Fig. 10).

## Discussion

**Figure 11:**
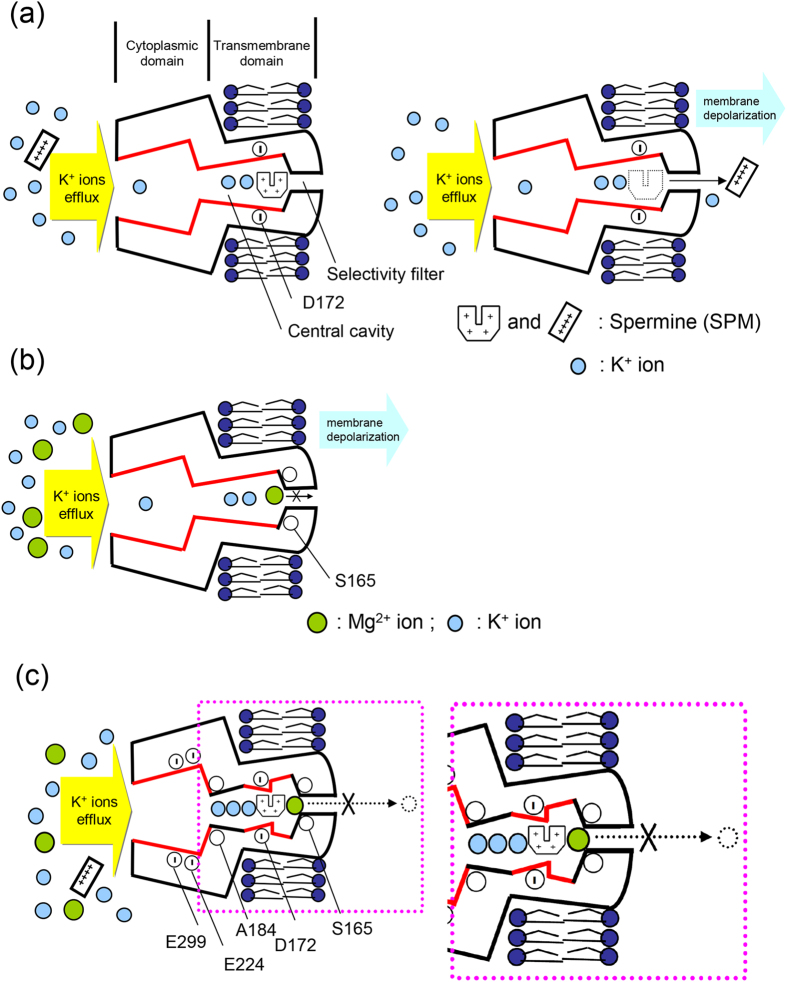
A Schematic model illustrating the mechanism underlying Mg^2+^ block of the Kir2.1 channel pore. (**a**) The WT Kir2.1 channel comprises cytoplasmic and transmembrane domains. The ion conduction pathway in the transmembrane domain could be further divided into the selectivity filter and the central cavity. SPM probably binds to a blocking site involving D172 with a curly form^13^. Moreover, SPM may go through the selectivity filter of the Kir2.1 channel, entering the extracellular side of the channel if there is a large driving force. (**b**) With strong outward K^+^ currents, intracellular Mg^2+^ is pushed to the outermost site of the flux–coupling region (probably involving S165) in the Kir2.1 channel pore. Mg^2+^ is more weakly bound to its site than SPM. However, unlike SPM, intracellular Mg^2+^ could not traverse the selectivity filter of Kir2.1 channel pore and exit to the outside even with a large driving force. (**c**) We illustrate that concomitant intracellular Mg^2+^ and SPM are synergistic in the blocking action of the Kir2.1 channel pore. SPM has a strong flow–dependent movement in the central cavity, and thus a strong blocking effect on the outward K^+^ currents when SPM gets “stuck” at the junction between central cavity and selectivity filter. SPM, however, can not completely block the outward K^+^ currents even with highly preponderant outward K^+^ flux (e.g., very strong depolarization) because of the possibility of outward exit of the blocking SPM through the selectivity filter. On the other hand, Mg^2+^ has a much weaker flow–dependent movement in the central cavity, and thus millimolar Mg^2+^ has an even weaker overall blocking effect on the outward K^+^ currents than micromolar SPM. Concomitant Mg^2+^ and SPM, however, would have a synergistic rather than additive blocking effect on the outward K^+^ current especially at strong depolarization. This is because the apparently weaker blocker Mg^2+^ could prohibit the outward exit of SPM and thus effectively reduce the residual outward K^+^ currents, either by direct physical hindrance or by the possible narrowing conformational changes at ~D172 residues induced by coexisting Mg^2+^ and SPM (Fig. 10), or by both.

We have seen that intracellular Mg^2+^ blocks the Kir2.1 channel pore in both voltage– and flow– dependent manners (Figs 1 and 2). Also, the blocking effect and especially the flow–dependence is markedly abolished by specific mutations involving E224, E299, and the bundle–crossing region (I176–A184), with double mutations either offsetting the effect of each single mutation or showing a strong negative cooperative effect (Figs 5, 6, 7). Mg^2+^ therefore very likely has its inward rectifying blocking effect on the Kir2.1 channel based on the same flux–coupling pore region as SPM, except that the Mg^2+^ blocking site probably is located closer to S165 than D172 with preponderant K^+^ efflux (Fig. 10 and supplementary Figure S2). It is interesting that Mg^2+^, an alkali earth metal cation with a very small crystal ionic radius and 2 charges (and thus a very high charge density), should have its blocking effect roughly at the same pore segment as SPM, presumably a long molecule with 4 regularly space positive charges and 3 intervening hydrophobic segments. The fact that the two very different blockers act on the same region of the pore would strongly indicate that functionally there is only one flux–coupling region responsible for the flow–dependent blocking phenomenon of the Kir2.1 channel pore. Moreover, although this region very likely contains regularly or semi–regularly spaced ionic sites and intervening hydrophobic groups in view of the very high IR index of SPM, it probably is not a fixed narrow fit for the permeating ions (a configuration one may originally imagine for a pore segment showing prominent flux–coupling phenomenon). Instead, it caliber could be so wide that Mg^2+^ could also cross the hydrophobic segment between two ionic sites “rapidly” enough to make a discernible flux–coupling blocking effect (see below). The initial very fast phase followed by a much slower phase of the “slow tails” upon repolarization in the presence of either SPM or Mg^**2+**^ (Figs 3 and 5,^12^) would also support a caliber wide enough to allow ionic passage at a somehow “blocked” segment of the pore (Fig. 11).

Although Mg^2+^ and SPM block the same flux–coupling segment of the Kir2.1 channel pore at the bundle–crossing region (Figs 5, 6, 7,^12^,^13^) with qualitatively similar features, there is a prominent quantitative difference in flow dependence (~800–fold difference in the IR index, Fig. 1^12^,^13^). The “perfectness” of flux–coupling block would be very much dependent on the “completeness” of coupled movement of ions between the ionic sites in the segment. The extremely high IR index of SPM therefore signals 4 or more sequential ionic sites with intervening hydrophobic parts each 5–6 Å in length, so that the configuration of the set of sites perfectly matches that of SPM. These sites probably are more or less spirally arranged in space; in view that only a short sequence (D172–A184) from each subunit is directly responsible for this pore region. Mg^2+^ does not have 4 separate charged groups connected in a row. In addition, because of its higher charge density than SPM, it may tend to face a higher energy barrier while crossing the intervening hydrophobic segments. The slower movement of Mg^2+^ between ionic sites (than SPM) may also contribute to the much smaller flow–dependence of block. On the other hand, it is intriguing that the intrinsic voltage–dependence of approaching and leaving the flux–coupling segment is rather similar for intracellular SPM and Mg^2+^ (Fig. 4^12^). In other word, the very asymmetrical energy barrier (in terms of electrical distance) at the bundle–crossing point or the “gate” of the pore remains similar for very dissimilar ions such as Mg^2+^ and SPM. The molecular substrate of the asymmetry of the barrier therefore is more likely “structural” (e.g., asymmetry in pore caliber or geometry) than “functional” (e.g., asymmetry in reorientation of the permeating ions or coordinating ligands). In other words, the bundle–crossing point (~A184^12^,^13^) is a significant energy barrier for ion permeation because of its small caliber (probably the second narrowest part of the pore after the selectivity filter). Moreover, there is very likely a much abrupt decrease in caliber from cytoplasmic domain than from the central cavity to this point (Fig. 11). These functional pictures are not entirely revealed by the crystallographic study^24^,^25^,^29^,^30^, but may reveal interesting rationales of the structure–function design of the Kir2.1 channel pore. The central cavity is designed for the inward rectifying block of SPM, but not so specific (so narrow and tightly fit) for SPM as to make Mg^2+^ another weaker inward rectifying blocker of the channel. The flux–coupling movement of permeating and blocking ions would be less accentuated toward the outermost point of this cavity because of the larger caliber. This may allow a small but definite chance that the blocker does not stay at the outermost position in the cavity with small outward K^+^ currents (small outward driving force, the actual scenario in the resting physiological conditions). A small outward K^+^ conductance may therefore persist to significantly contribute to keep the resting membrane potential close to the K^+^ reversal potential, a basic physiological function of the Kir channels (see below).

We have seen that SPM, even with its much lower physiological concentrations (~10 μM^8^,^14^,^15^,^16^,^17^) taken into consideration, is a much stronger inward rectifying blocker than Mg^2+^ (Figs 1, 2 and 8,^12^,^13^). However, 10 μM SPM can not fully block the outward currents even at strong depolarization (Fig. 8). This is partly ascribable to the possibility of outward exit of SPM from the pore^12^,^27^. In contrast, it is essentially impossible for internal Mg^2+^ to traverse the selectivity filter and exit the pore outwardly. Concomitant Mg^2+^ therefore may further reduce of the residual outward K^+^ currents in the presence of SPM at strong depolarization (Fig. 8), either directly or via associated conformational changes of the pore, or both (Fig. 10). Mg^2+^, with its physiological intracellular concentration in the millimolar range, may also help to bring down the outward K^+^ currents almost “immediately” upon membrane depolarization. Intracellular Mg^2+^ therefore well serves as an adjunct blocker to SPM to assure little outward K^+^ currents through the Kir2.1 channel during the entire depolarization phase. On the other hand, neither SPM nor Mg^2+^ could so effectively block outward K^+^ currents when the membrane is repolarized to near the K^+^ reversal potential, because the blockers will have an increased tendency and rapid movement (e.g., the very fast phase of the slow tails) to stay in the widened middle part of the central cavity due to the decreased preponderance of outward K^+^ flux (despite of different degree of preponderance, there would always be preponderant outward K^+^ flux in physiological conditions because the membrane potential can not go below the K^+^ reversal potential). Intracellular Mg^2+^ and SPM (or polyamine) and the Kir channels in the cell membrane would therefore make an ideal combination to assure not only essentially undisturbed initiation of cellular activities (irrespective of the rising speed and duration of the action potentials) but also rapid “stabilization” of the membrane as soon as the intervening repolarization phase ensues.

## Methods

### *in vitro* PCR mutagenesis and molecular biology techniques

The mouse Kir2.1 DNA was subcloned into phagemid expression vector of Bluescript II SK1^12^,^13^,^27^,^31^. All mutations were inserted into the plasmid encoding WT Kir2.1 DNA using the QuikChange II XL site–directed mutagenesis kit (Stratagene, LA Jolla, CA, USA), and all mutant cDNA were confirmed by DNA sequencinger (Applied Biosystems, 3730Xl DNA, Analyzer Foster, CA, USA). These plasmids containing Kir2.1 DNA were linearized with restriction enzyme digestion as described previously^12^,^13^,^27^. The full–length complementary RNA (cRNA) transcript was synthesized from purified linearized cDNA with T7 polymerase transcription reactions (*in vitro* T7 mMESSAGE mMACHINE transcription kits, Ambio, Austin, TX, USA).

### Preparation of *Xenopus* oocytes

All animal handling procedures were approved by the Institutional Animal Care and Use Committee (IACUC) of National Taiwan University College of Medicine (Permit number: 2006–2320), and were performed in accordance with animal welfare guidelines by the National Institutes of Health (NIH)^12^,^13^,^27^. *Xenopus* oocytes (stages V and VI) were isolated by partial ovariectomy from frogs anaesthetized with 0.1% (w/v) tricaine (3–aminobenzoic acid ethyl ester), defolliculated using 1.5% type IA collagenase (Sigma Chemical Co., St. Louis, MO, USA) and then maintained at 18 °C in ND 96 solution which contains 96 mM NaCl, 5 mM HEPES, 2 mM KCl, 1.8 mM MgCl_2_, 1.0 mM CaCl_2_, titrated to pH 7.6 by 1 M NaOH. *Xenopus* oocytes were microinjected 40–60 nl wild–type (WT) or mutant cRNA (Nanojector II, Drummond Scientific Inc, Japan) and were maintained in ND 96 solution at 18 °C for no longer than 7 days after defolliculation^12^,^13^,^27^. The *Xenopus* oocyte was then used for recording right after wash in the external solution containing: 72 mM KCl, 2 mM KH_2_PO_4_, 8 mM K_2_HPO_4_, and 5 mM K_2_EDTA, titrated to pH 7.6 by 1 M KOH.

### **Excised inside–out giant patch recordings in** ***Xenopus*** **oocyte**

Excised inside–out giant patch recordings from *Xenopus* oocytes expressing WT or mutant Kir2.1 channels were conducted at room temperature (22–25 °C) using the pClamp 6.0 software and an Axopatch 200A amplifier (Axon Instruments, Inc. Sunnyvale, CA, USA). Data were filtered at ~2 kHz and digitized at ~10 kHz through Digidata 1200A interface (Axon Instruments, Inc. Sunnyvale, CA, USA). The pipettes were pulled from borosilicate glass (Warner Instruments, MA, USA) using a horizontal puller (Zeitz Instruments, Inc. Martinsried, Germany) and fired polished (Narishige scientific instruments, Inc, Japan). The pipette resistance was 0.2–0.5 MΩ when filled with extracellular solutions. Both the extracellular and intracellular solutions contained (the 100 mM K^+^ solution): 68 mM KCl, 8 mM K_2_HPO_4_, 2 mM KH_2_PO_4_, 5 mM K_2_EDTA, and 4 mM KOH, titrated to pH 7.4 by 1 M KOH^12^,^13^,^27^. For the experiments in symmetrical 20 mM K^+^, intracellular and extracellular solutions both contained: 8.6 mM KCl, 4 mM K_2_HPO_4_, 1 mM KH_2_PO_4_, 3 mM EDTA, and 2.4 mM KOH, titrated to pH 7.4 by 1 M KOH. For the experiments in symmetrical 300 mM K^+^, intracellular and extracellular solutions both contained: 270 mM KCl, 8 mM K_2_HPO_4_, 2 mM KH_2_PO_4_, 5 mM K_2_EDTA, and 2 mM KOH, titrated to pH 7.4 by 1 M KOH. The desired total concentration of Mg^2+^ was obtained by mixing an appropriate volume of 5 mM Mg^2+^ intracellular solution with Mg^2+^–free intracellular solution, with free concentration of Mg^2+^ calculated by the WinMAXC32 software (Chris Patton, Stanford University, USA)^15^,^26^. For the experiments in 20 mM external K^+^, the intracellular solution was not changed, but the extracellular solution contained 20 mM K^+^ (see above for the detailed component of the solution). The excised inside–out giant patch was moved in front of an array of square glass (Warner Instruments, Inc. Hamden, CT, USA) emitting different intracellular solutions, and was always subject to continuous perfusion of the intracellular solution for at least 10 min to wash out endogenous intracellular blockers before actual experiments were carried out.

## Data analysis

Data were analyzed using Clmapfit 9.0 (Axon Instruments, Inc. Sunnyvale, CA, USA), and Sigmaplot 10.0 software (Systat software, Inc. Germany)^12^,^13^,^27^. The susceptibility of WT and mutant channels to intracellular Mg^2+^ has been examined based on the apparent dissociated constant (Kd) values from the Hill’s equation (one–to–one binding reaction)^15^,^26^,^32^,^33^,^34^. In this study, the dose–dependent blocking effect of intracellular Mg^2+^ could be reasonably fitted with a simplest form of Hill’s equation at membrane potentials more positive than ~−60 mV in symmetrical 100 mM K^+^ or more positive than −100 mV in 20 mM external/ 100 mM internal K^+^ (Eq. 1):


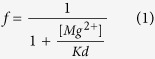


where Kd is the apparent dissociation constant of Mg^2+^, [Mg^2+^] is the concentration of Mg^2+^, and f (the “relative currents”) is the ratio between the steady–state current amplitude in Mg^2+^ and that in control. All data are presented as mean ± SEM. Statistical significance was assessed using Student’s independent *t* test.

### Homology modeling and molecular dynamics simulation of Kir2.1 channel pore

The modeling and simulation were performed in a similar way to that described in previous studies^12^,^13^,^27^. Briefly, the X–ray crystallographic structure of the chicken Kir2.2 (PDB ID: 3SPI) and mouse Kir2.1 (PDB ID: 1U4F) were used as templates for building the homology models of mouse Kir2.1 channel^24^,^25^,^29^,^30^. Homology modeling of the mouse Kir2.1 channel was done with Discovery Studio V3.0 (DS V3.0) Client programs (Accelrys Inc., San Diego, CA, USA)^12^,^13^,^27^. The molecular dynamic simulation of the Kir2.1 channel was performed by Chemistry at HARvard Molecular Mechanics (CHARMM) force field and DS V3.0 programs (Accelrys Inc., San Diego, CA, USA)^12^,^13^,^27^. Constant temperature and pressure were applied with 300 K° and 1 bar^35^. In preparation of the simulation, counter–ions were applied to ensure that the net charge on the molecular dynamics simulation system was zero. Also, K^+^ ions and water molecules were free to move during the energy minimization, heating, equilibrium and production process^12^,^13^,^27^. In the presence of approximately forty water molecules, seven K^+^ ions and/or one Mg^2+^ or one SPM molecule were applied to the central cavity region of Kir2.1 channel, and the one with the lowest potential energy were selected from approximately twenty candidate models.

## Additional Information

**How to cite this article**: Chiung–Wei, H. and Chung–Chin, K. A synergistic blocking effect of Mg^2+^ and spermine on the inward rectifier K^+^ (Kir2.1) channel pore. *Sci. Rep.* **6**, 21493; doi: 10.1038/srep21493 (2016).

## Supplementary Material

Supplementary Information
