# Supplementary material for: A synergistic blocking effect of Mg2+ and spermine on the inward rectifier K+ (Kir2.1) channel pore
Source: Sci Rep. 2016 Feb 12;6:21493. doi: 10.1038/srep21493 (PMC4751470; doi:10.1038/srep21493)
Supplement: Supplementary Information [file srep21493-s1.doc]

**Supplementary information**

A synergistic blocking effect of Mg^2+^ and spermine on the inward rectifier K^+^ (Kir2.1) channel pore

**Chiung–Wei Huang ^1^ and Chung–Chin Kuo ^1, 2^**

1. Department of Physiology, National Taiwan University College of Medicine, Taipei, Taiwan
2. Department of Neurology, National Taiwan University Hospital, Taipei, Taiwan

Correspondence should be addressed:

**Chung**–**Chin Kuo**

Department of Physiology, National Taiwan University College of Medicine,

No. 1, Jen–Ai Road, 1st Section, Taipei 100, Taiwan.

Tel: (886)–2–23123456 ext 88236

Fax: (886)–2–23964350

E–mail: [chungchinkuo@ntu.edu.tw](mailto:chungchinkuo@ntu.edu.tw)

**Figure and legend**

**Figure S1**

**Figure S1 Lack of intracellular Mg^2+^ and SPM on the non**−**Kir2.1 channel expressing membranes in symmetrical 100 mM K^+^**

There is no discernible currents, nor do intracellular Mg^2+^, SPM and concomitant Mg^2+^ and SPM show any evident on a non−Kir2.1−expressing membrane patch in symmetrical 100 mM K^+^ (the same protocol as that in the Figure 1a).

**Figure S2**

**Figure S2 Inhibition of the S165L and D172N mutant Kir2.1 currents by intracellular Mg^2+^**

The change in Kd around the reversal potential in the Kd–voltage plot is less dramatic in the D172N and S165L mutant than in the WT channels. The IR indices (the ratio between Kd values at –30 and +30 mV in symmetrical 100 mM K^+^) in the S165L and D172N mutant are ~14 and ~25, respectively. The WT data (indicated by the solid line) are taken from Figure 1c for comparison.
